# Steroid responsiveness in alcohol-associated hepatitis is linked to glucocorticoid metabolism, mitochondrial repair, and heat shock proteins

**DOI:** 10.1097/HC9.0000000000000393

**Published:** 2024-03-04

**Authors:** Josiah Hardesty, Meghan Hawthorne, Le Day, Jeffrey Warner, Dennis Warner, Marina Gritsenko, Aliya Asghar, Andrew Stolz, Timothy Morgan, Craig McClain, Jon Jacobs, Irina A. Kirpich

**Affiliations:** 1 Department of Medicine, Division of Gastroenterology, Hepatology, and Nutrition, University of Louisville, Louisville, Kentucky, USA; 2 Department of Pharmacology and Toxicology, University of Louisville School of Medicine, Louisville, Kentucky, USA; 3 Department of Biological Sciences, Biological Sciences Division and Environmental Molecular Sciences Laboratory, Pacific Northwest National Laboratory, Richland, Washington, USA; 4 Department of Medicine and Research Services, Medicine and Research Services, VA Long Beach Healthcare System, Long Beach, California, USA; 5 Department of Medicine, Division of Gastrointestinal and Liver Disease, Keck School of Medicine, University of Southern California, Los Angeles, California, USA; 6 Robley Rex Veterans Medical Center, Louisville, Kentucky, USA; 7 Department of Medicine, University of Louisville Alcohol Center, University of Louisville School of Medicine, Louisville, Kentucky, USA; 8 Department of Medicine, University of Louisville Hepatobiology and Toxicology Center, University of Louisville School of Medicine, Louisville, Kentucky USA; 9 Department of Microbiology and Immunology, University of Louisville School of Medicine, Louisville, KY, USA

## Abstract

**Background:**

Alcohol-associated hepatitis (AH) is one of the clinical presentations of alcohol-associated liver disease. AH has poor prognosis, and corticosteroids remain the mainstay of drug therapy. However, ~40% of patients do not respond to this treatment, and the mechanisms underlying the altered response to corticosteroids are not understood. The current study aimed to identify changes in hepatic protein expression associated with responsiveness to corticosteroids and prognosis in patients with AH.

**Methods:**

Patients with AH were enrolled based on the National Institute on Alcohol Abuse and Alcoholism inclusion criteria for acute AH and further confirmed by a diagnostic liver biopsy. Proteomic analysis was conducted on liver samples acquired from patients with AH grouped as nonresponders (AH-NR, n = 7) and responders (AH-R, n = 14) to corticosteroids, and nonalcohol-associated liver disease controls (n = 10). The definition of responders was based on the clinical prognostic model, the Lille Score, where a score < 0.45 classified patients as AH-R and a score > 0.45 as AH-NR. Primary outcomes used to assess steroid response were Lille Score (eg, improved liver function) and survival at 24 weeks.

**Results:**

Reduced levels of the glucocorticoid receptor and its transcriptional co-activator, glucocorticoid modulatory element-binding protein 2, were observed in the hepatic proteome of AH-NR versus AH-R. The corticosteroid metabolizing enzyme, 11-beta-hydroxysteroid dehydrogenase 1, was increased in AH-NR versus AH-R along with elevated mitochondrial DNA repair enzymes, while several proteins of the heat shock pathway were reduced. Analysis of differentially expressed proteins in AH-NR who survived 24 weeks relative to AH-NR nonsurvivors revealed several protein expression changes, including increased levels of acute phase proteins, elevated coagulation factors, and reduced mast cell markers.

**Conclusions:**

This study identified hepatic proteomic changes that may predict responsiveness to corticosteroids and mortality in patients with AH.

## INTRODUCTION

Alcohol-associated liver disease (ALD) is an ever-growing societal burden resulting in untimely death^1^ and associated with increased health care expenditures.^2^ ALD is a spectrum of liver pathology, which ranges from steatosis to steatohepatitis, to cirrhosis with various degrees of fibrosis, and potentially to HCC.^3^ Alcohol-associated hepatitis (AH) is the clinical presentation within the continuum of ALD that can occur in patients with or without underlying cirrhosis. Patients with AH can present with jaundice, liver failure, systemic inflammatory response syndrome, and sepsis,^4^ all of which contribute to patient mortality.^5^ The 30-day mortality rate for severe AH can be as high as 20%–30%.^2,6^

The most common treatment regimen for AH is corticosteroids (eg, prednisolone or prednisone—glucocortoid receptor agonists with anti-inflammatory effects), with the duration of treatment based on patient response.^7,8^ The response to corticosteroid treatment is identified based on the Lille Score (a prognostic model to evaluate therapeutic response to corticosteroids for patients with AH^8^), and treatment is discontinued if a patient is determined to be a “nonresponder” at day 7.^2,8^ Thus, a Lille Score of ≤ 0.45 was shown to predict a positive response to corticosteroids, whereas a Lille Score of > 0.45 indicates no improvement in liver function and that this treatment regimen should be stopped.^2,8^ Other therapies for AH have been evaluated, such as pentoxifylline, which is an anti-inflammatory drug that reduces the synthesis of TNF-α and has fewer side effects as compared to corticosteroids. However, the STOPAH trial demonstrated no impact on mortality by pentoxifylline in patients with AH.^9^ Alternative therapies, including Anakinra,^10^ G-CSF,^11,12^ and other TNF inhibitors,^7^ have also been investigated but have shown limited effects in the treatment of AH. Notably, not all patients with AH respond to corticosteroids, as assessed by the Lille Score at day 7. Nonresponders to corticosteroids have more frequent hepatic ballooning degeneration,^13^ increased susceptibility to infections,^14^ and increased short-term mortality.^8^ The reasons/mechanisms for corticosteroid nonresponse are not understood. This current study aimed to identify hepatic proteomic differences between AH responders (AH-R) to corticosteroid treatment and AH nonresponders (AH-NR) and to evaluate differences in the liver proteome associated with survival in AH-NR patients. This is an extension of our previously published work investigating the changes in the liver proteome in patients with AH^15^ focused on describing the proteome differences associated with response to corticosteroid treatment.

## METHODS

### Study populations and clinical characterization

This study is an extension of our published work,^15^ wherein we analyzed the proteome from liver biopsy samples obtained from patients with AH before treatment. In the current study, we focused on the subset of patients (n = 21) treated with glucocorticoids (prednisolone (n = 17) or prednisone (n = 4). Liver biopsies obtained from non-ALD subjects (n = 10) were acquired from the Liver Tissue Cell Distribution System at the University of Minnesota (NIH contract HHSN276201200017C). The Liver Tissue Cell Distribution System confirmed no underlying pathology for these samples. Patients with AH were selected to receive steroid treatment based on the National Institute on Alcohol Abuse and Alcoholism inclusion criteria for acute AH.^16^ Patients with AH were administered corticosteroids within 2–3 days of interpretation of the diagnostic liver biopsy by the attending physician. Clinical response to prednisolone or prednisone was determined by Lille Score, which was calculated on day 7 of corticosteroid treatment. A Lille Score < 0.45 was used to determine a positive response to treatment, while a Lille Score > 0.45 indicated no response. Based on these scores, patients were divided into 2 groups: responders (AH-R, n = 14) and nonresponders (AH-NR, n = 7). Lille Score was used to classify responders since the identification of mechanisms of corticosteroid response was the primary goal of this study. To better identify markers of mortality, AH-NR were further divided based on survival at 24 weeks postadmission into survivors (AH-NR-S, n = 3) and nonsurvivors (AH-NR-NS, n = 4). Recently, in patients with AH, mortality within 24 weeks of diagnosis was found to be more closely linked to liver injury,^17^ which is why we chose this timepoint for this comparison. All study protocols conformed to the ethical guidelines of the 1975 Declaration of Helsinki^18^ and the Institutional Review Board (IRB Protocol# 1411). All participating patients provided informed consent for the initial clinical study. Liver specimens were not acquired from executed prisoners or institutionalized persons.

### Liver proteome analyses

Liver proteomic analyses were conducted in the Pacific Northwest National Laboratory using standard protocols and analyzed as described^15^ and briefly summarized below. These data were deposited in the MassIVE repository under the accession number MSV000089168. Liver tissues were homogenized in protein extraction buffer supplemented with protease and phosphatase inhibitors, followed by centrifugation and reduction by dithiothreitol and alkylation with iodoacetamide. Protein was enzymatically digested with Lys-C followed by desalting of the peptides and concentration through a Savant Speed-Vac concentrator (Thermo Fisher Scientific, Waltham, MA). Isolated peptide sample concentrations were measured, followed by metabolic labeling with TMT labels. TMT-labeled peptides were then fractionated before LC/MS/MS analysis on the Q Exactive HF Hybrid Quadrupole-Orbitrap mass spectrometer (Thermo Fisher Scientific, Waltham, MA).

### Plasma mtDNA measurement

Intact mitochondrial DNA (mtDNA) was measured in matched plasma samples from AH-R and AH-NR patients through the Mitochondrial DNA Damage Assay Kit (Detroit R&D, Detroit, MI).

### Statistical analysis

All continuous variables are presented as mean±SEM. Data between 2 groups were compared by unpaired Student *t*-test, and data between multiple groups were compared by one-way ANOVA using InfernoRDN software (available at https://www.pnnl.gov/integrative-omics, last accessed January 10, 2023). Principal component analysis was conducted in GraphPad Prism (version 9.5.0, San Diego, CA). Principal component scores were then visualized via RStudio Software (version 1.3.1093, Boston, MA) using the plot3D function of the rgl package.^19^ A *p* < 0.05 was considered significant for all statistical tests.

## RESULTS

### Characterization of patient population

**FIGURE 1:**
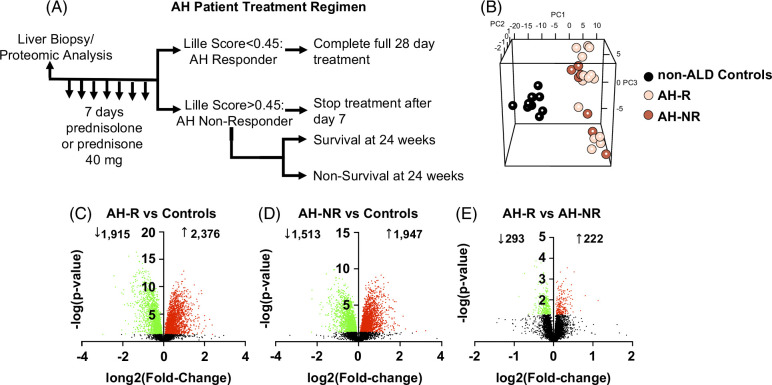
Hepatic proteomic differences between non-ALD controls, AH-R, and AH-NR groups. (A) Study design for hepatic proteomic analysis of liver tissue acquired from AH-R and AH-NR patients who received corticosteroid therapy. (B) PCA of non-ALD control (black), AH-R (blue), and AH-NR (red) hepatic proteome. Volcano plots highlighting significantly increased (red) and decreased (green) proteins in (C) AH-R versus controls, (D) AH-NR versus controls, and (E) AH-R versus AH-NR. *p* < 0.05 was considered significant. Abbreviations: AH, alcohol-associated hepatitis; AH-R, AH responders; AH-NR, AH nonresponders; ALD, alcohol-associated liver disease; PCA, principal component analysis.

Patients that met the National Institute on Alcohol Abuse and Alcoholism inclusion criteria for acute AH^16^ were enrolled in the study. After the patient liver biopsy confirmed AH (eg, 2–3 days after biopsy), the attending physician initiated the corticosteroid regimen for the patients with AH. Patients were administered either prednisolone or prednisone at 40 mg per day for 7 days. Based on Lille Score, patients were classified as either AH-R or AH-NR (Figure 1A). Treatment was discontinued in nonresponders while responders continued the treatment course for a total of 28 days. Based on mortality at 24 weeks, AH-NR patients were further divided into survivors (AH-NR-S) and nonsurvivors (AH-NR-NS). Patient demographics and select clinical parameters are summarized in Table 1. The study cohort consisted of 18 males and 3 females across AH-R and AH-NR patients and 10 males in the non-ALD group. AH-R patients were significantly younger than non-ALD controls and AH-NR patients. All of the AH-NR and 10 of the AH-R patients were treated with prednisolone while 4 AH-R patients were treated with prednisone. Within the AH-NR cohort, there were no significant differences in age, Model for End-Stage Liver Disease, or Lille Score in AH-NR-S versus AH-NR-NS.

**TABLE 1:** Provides patient demographic data for non-ALD controls, AH-R, AH-NR, and AH-NR who survived or did not survive 24 weeks

	Non-ALD controls (n = 10)	AH-R (n = 14)	AH-NR (n = 7)	AH-NR-S (n = 3)	AH-NR-NS (n = 4)
Age	56 ± 2.7	37.6 ± 2.7^a^	51 ± 4.4^b^	51 ± 9.9	50.8 ± 4.2
Sex	10M	12M/2F	6M/1F	3M	3M/1F
MELD	N/A	26.1 ± 1.4	23.6 ± 0.9	23 ± 0.88	24.5 ± 1.4
Lille Score	N/A	0.205 ± 0.04	0.761 ± 0.06^b^	0.710 ± 0.07	0.828 ± 0.14
Treatment (n)	N/A	Prednisolone (10) Prednisone (4)	Prednisolone (7)	Prednisolone (3)	Prednisolone (4)

*Notes:* Statistical significance (*p* < 0.05) was denoted by the following characters for the given comparison.

a vs controls.

b vs AH-R.

Abbreviation: AH-NR-NS, AH nonresponder non-survivors; ALD, alcohol-associated liver disease; MELD, Model for End-Stage Liver Disease.

### Hepatic proteomic differences between non-ALD controls and the AH-R and AH-NR groups

Principal component analysis showed clear separation in hepatic proteome between non-ALD controls and patients with AH but did not reveal distinct differences between the AH-R and AH-NR cohorts (Figure 1B). This suggests that AH-R and AH-NR have very similar hepatic proteomes before treatment and that distinct protein expression changes differentiate the 2 groups as opposed to global protein expression patterns. Compared with non-ALD controls, 4291 (1915 decreased, and 2376 increased) proteins were differentially expressed in AH-R (Figure 1C), and 3460 (1513 decreased, and 1947 increased) in AH-NR (Figure 1D). A comparison between AH-R and AH-NR revealed a total of 515 proteins (293 decreased and 222 increased) that were significantly changed (Figure 1E). The full proteomic data can be found in Supplemental Table S1, http://links.lww.com/HC9/A839.

To identify hepatic markers associated with responsiveness or nonresponsiveness to corticosteroid treatment in patients with AH, we performed the Gene Ontology process analysis, which identified multiple pathways that were altered in AH-R and AH-NR groups (Supplemental Fig. S1, http://links.lww.com/HC9/A829).

### Glucocorticoid metabolism and signaling are altered in AH-NR relative to AH-R

**FIGURE 2:**
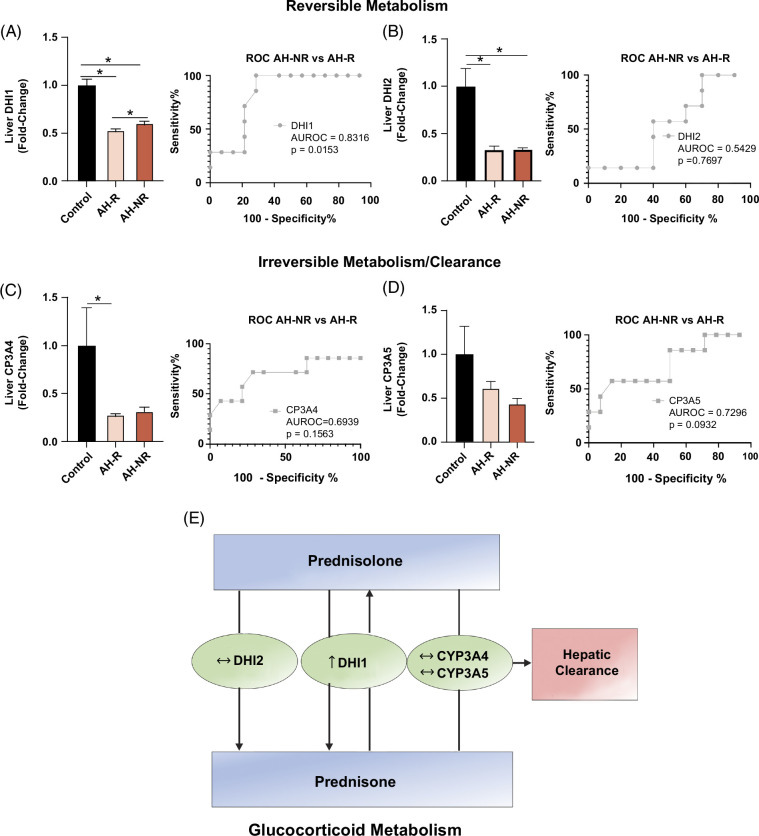
Reversible glucocorticoid metabolism is increased in AH-NR compared to AH-R. Protein expression and the associated AUROC curves for (A) DHI1, (B) DHI2, (C) CP3A4, and (D) CP3A5. (E) Summary of corticosteroid metabolism in AH-NR versus AH-R. Data are presented as mean ± SEM. *p < 0.05 was considered significant. Abbreviations: AH, alcohol-associated hepatitis; AH-R, AH responders; AH-NR, AH nonresponders; CYP3A4, cytochrome P450 3A4; CYP3A5, cytochrome P450 3A5; DHI1, 11-beta-hydroxysteroid dehydrogenase 1; DHI2, 11-beta-hydroxysteroid dehydrogenase 2.

As the aim of the study was to identify possible processes and mechanisms contributing to nonresponse to corticosteroid treatment, the 515 significantly changed proteins in AH-NR versus AH-R were further evaluated for any differences in corticosteroid metabolism and signaling. We observed that 11-beta-hydroxysteroid dehydrogenase 1 (DHI1), a bidirectional enzyme that reversibly metabolizes active prednisolone to the less active form, prednisone, was upregulated in AH-NR compared to AH-R. Furthermore, DHI1 expression could distinguish AH-NR from patients with AH-R (Figure 2A). 11-beta-hydroxysteroid dehydrogenase 2, which also metabolizes prednisolone to prednisone, was not significantly different between AH-NR and AH-R (Figure 2B), as well as the expression of other enzymes involved in irreversible phase I hepatic metabolism and clearance of prednisolone, such as cytochrome P450 3A4 and 3A5 (Figure 2C-D). Figure 2E summarizes changes in the corticosteroid metabolism pathway in AH-NR versus AH-R.

**FIGURE 3:**
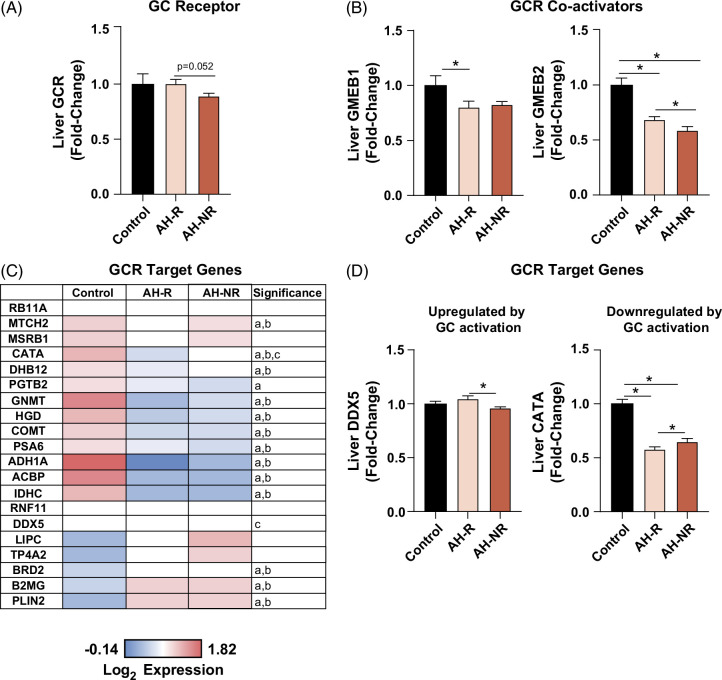
Glucocorticoid signaling is compromised in AH-NR relative to AH-R. Protein expression for (A) GCR and (B) GMEB1 and GMEB2. (C) Heatmap of GCR target gene expression at the protein level. Protein expression for (D) DDX5 and CATA. Data are presented as mean ± SEM. *p < 0.05 was considered significant. The following letters denote significance a: AH-R versus control, b: AH-NR versus control, and c: AH-NR versus AH-R. Abbreviations: AH, alcohol-associated hepatitis; AH-R, AH responders; AH-NR, AH nonresponders; CATA, catalase; DDX5, DEAD box protein 5; GCR, glucocorticoid receptor; GMEB1, glucocorticoid modulatory element-binding protein 1; GMEB2, glucocorticoid modulatory element-binding protein 2.

Given that prednisolone acts through the glucocorticoid receptor (GCR), we next evaluated the expression of GCR in our cohorts. Compared to controls, the expression of GCR was not changed in AH-R but was downregulated in AH-NR, but more importantly, it was lower in AH-NR versus AH-R (*p* = 0.052) (Figure 3A). The expression of the GCR transcriptional co-activator, glucocorticoid modulatory element-binding protein 1 was reduced in both AH cohorts versus control (being significant only for the AH-R versus control comparison) and was similar between AH-NR and AH-R. The expression of another co-activator, glucocorticoid modulatory element-binding protein 2 expression was significantly decreased in both AH groups versus control, and there was also a significant difference between AH-NR versus AH-R (Figure 3B). Analysis of hepatic GCR target genes revealed 2 clusters of proteins that were increased or decreased in AH compared to controls (Figure 3C). Among the GCR target genes, there were only 2 proteins that were significantly different between AH-NR versus AH-R, including DEAD box protein 5 (an RNA helicase) and catalase (Figure 3D).

### The heat shock response pathway was downregulated in AH-NR but not in AH-R relative to controls

**FIGURE 4:**
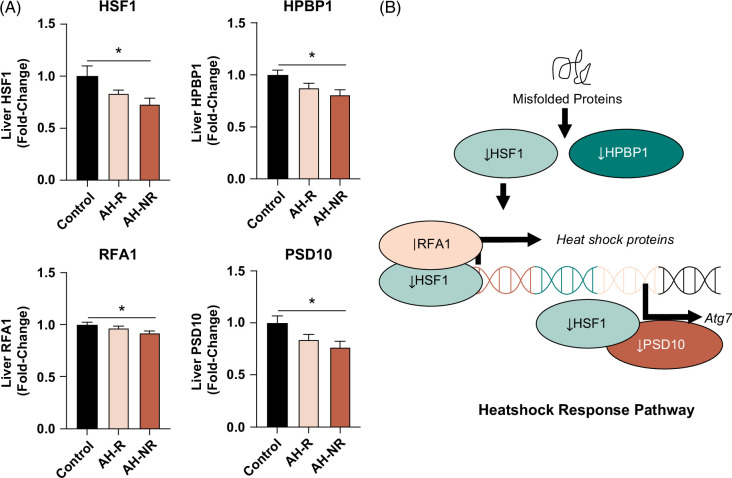
Heat shock response pathway was downregulated in AH-NR relative to non-ALD controls. (A) Protein expression of HSF1, HPBP1, RFA1, and PSD10. (B) Diagram of the heat shock response pathway in AH-NR versus Controls. Data are presented as mean ± SEM. *p < 0.05 was considered significant. Abbreviations: AH, alcohol-associated hepatitis; AH-R, AH responders; ALD, alcohol-associated liver disease; HPBP1, hsp70-binding protein 1; HSF1, heat shock factor 1; PSD10, 26S proteasome non-ATPase regulatory subunit 10; RFA1, replication factor A 70kDa DNA-binding subunit.

Misfolded protein aggregates (Mallory bodies) are commonly observed in hepatocytes from patients with AH^20^ and have been linked to compromised mechanisms of proteostasis.^21,22^ More recently, activation of heat shock factor 1 (HSF1) has been shown to be protective in models of ALD.^23^ In our study, heat shock response proteins were downregulated in AH-NR versus controls, including heat shock factor 1 (one of the regulators of the transcriptional response to misfolded proteins), hsp70-binding protein 1 (an HSF1 binding protein), and HSF1 transcriptional cofactors such as replication factor A 70kDa DNA-binding subunit, and the 26S proteasome non-ATPase regulatory subunit 10. (Figure 4A-B).

### Mitochondrial DNA repair proteins were upregulated in AH-NR but not in AH-R relative to controls

**FIGURE 5:**
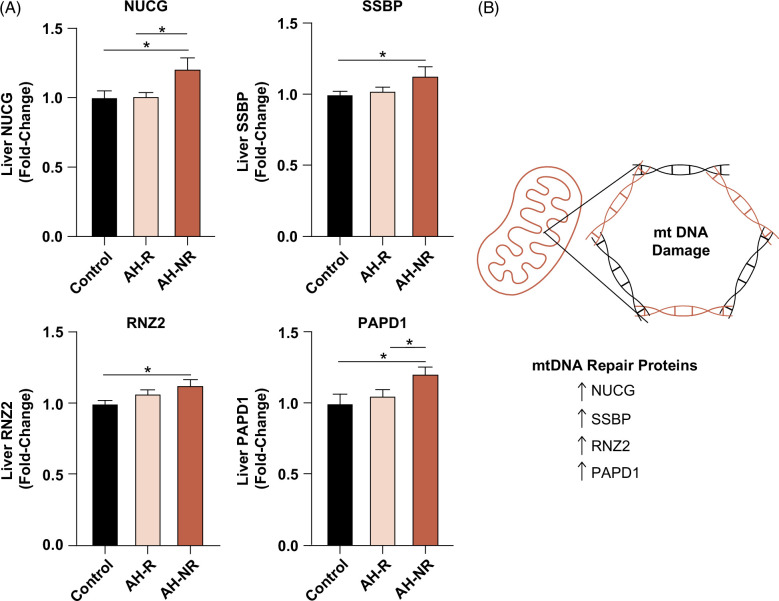
Mitochondrial DNA repair proteins were upregulated in AH-NR but not in AH-R relative to controls. (A) Protein expression of NUCG, SSBP, RNZ2, and PAPD1. (B) Summary of results in AH-NR versus controls where mtDNA repair proteins are upregulated. Data are presented as mean ± SEM. *p < 0.05 was considered significant. Abbreviations: mtDNA, mitochondrial DNA; NUCG, endonuclease G; PAPD1, poly(A) RNA polymerase 1; RNZ2, zinc phosphodiesterase ELAC protein 2; SSBP, single-stranded DNA-binding protein.

AH is known to be associated with mitochondrial dysfunction, including reactive oxygen species formation and mtDNA damage.^24^ There were 4 mitochondrial DNA repair proteins identified in our database that were upregulated in AH-NR but not in AH-R relative to controls, including mitochondrial endonuclease G, mitochondrial single-stranded DNA-binding protein, zinc phosphodiesterase ELAC protein 2, and mitochondrial poly(A) RNA polymerase (Figure 5A-B). Upregulation of these repair enzymes may be an adaptive response to mtDNA damage.^25^ However, intact plasma mtDNA, an indirect marker of hepatic mtDNA damage,^26^ was not significantly different between AH-R and AH-NR (Supplemental Fig. S2, http://links.lww.com/HC9/A829).

### Hepatic acute phase and coagulation factors were downregulated in AH-NR-NS, while mast cell markers were upregulated

**FIGURE 6:**
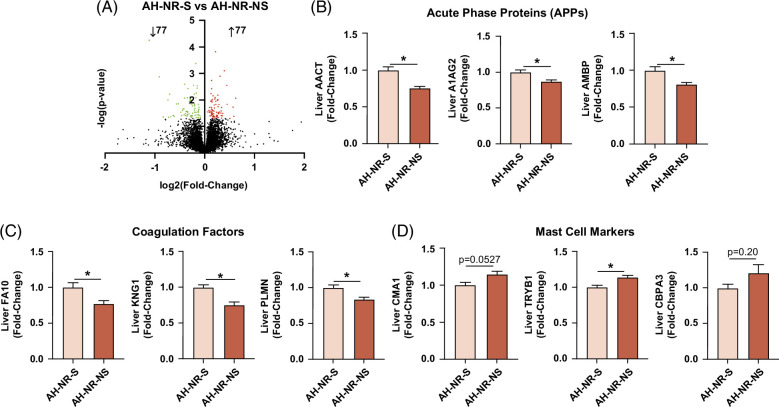
Hepatic acute phase proteins and coagulation factors were downregulated in AH-NR-NS, while mast cell markers were upregulated. (A) Volcano plot highlighting significant protein increased (red) and decreased (green) in AH-NR-NS versus AH-NR-S. (B) Expression of acute phase proteins, AACT, A1AG2, and AMBP. (C) Expression of coagulation factors, FA10, KNG1, and PLMN. (D) Expression of mast cell markers, CMA1, TRYB1, and CBPA3. Data are presented as mean ± SEM. *p < 0.05 was considered significant. Abbreviation: A1AG2, alpha-1-acid glycoprotein 2; AACT, alpha-1-antichymotrypsin; AH-NR-NS, AH nonresponder nonsurvivors; AH-NR-S, AH nonresponder survivors; AMBP, alpha-1-microglobulin; CMAI, chymase; CBPA3, carboxypeptidase A; FA10, coagulation factor X; KNG1, kininogen-1; PLMN, plasminogen; TRYB1, tryptase alpha/beta 1.

Mortality rates in individuals with severe AH range from 30% to 50%, with even higher rates in those that do not respond to corticosteroid treatment.^2,8^ Therefore, we sought to identify changes in basal hepatic protein expression that were associated with survivorship at 24 weeks for AH-NR patients. Within this cohort, there were 154 proteins with significant changes in expression (77 decreased, 77 increased) between the AH-NR-NS and AH-NR-S groups (Figure 6A). Among these proteins were several acute phase proteins (APPs), including alpha-1-antichymotrypsin, alpha-1-acid glycoprotein 2, and alpha-1-microglobulin, all of which were decreased in the AH-NR-NS group (Figure 6B). Further, there was also a significant decrease in the expression of the coagulation factors, kininogen-1, plasminogen, and coagulation factor X in AH-NR-NS versus AH-NR-S (Figure 6C). Conversely, the AH-NR-NS group had elevated markers of mast cells (pro-fibrotic immune cells^27^), including chymase, tryptase alpha/beta 1, and mast cell carboxypeptidase A (Figure 6D). These protein alterations indicate a greater degree of compromised liver function in AH-NR-NS patients, which could impact mortality. Full proteomic data for this comparison can be found in Supplemental Table S2, http://links.lww.com/HC9/A840.

## DISCUSSION

In this study, we identified hepatic proteomic differences in patients with AH associated with responsiveness or nonresponsiveness to steroid therapy and survival. Although AH-R and AH-NR were very similar before treatment based on their proteome as a whole, several specific protein expression changes were identified between the 2 groups. We found differences in the proteins involved in corticosteroid metabolism and signaling between responders and nonresponders to therapy. First, the expression of GCR was reduced in nonresponders versus responders, approaching statistical significance. Further, GCR co-activator, glucocorticoid modulatory element-binding protein 2, was significantly decreased in nonresponders versus responders. These data suggest that GCR signaling is more compromised in AH-NR versus AH-R. Downregulation of hepatic GCR may mediate nonresponsiveness to corticosteroids and could be a contributing factor to poor prognosis in AH-NR patients. A higher incidence of infection in AH-NR patients as compared to AH-R^14^ may also impact/reduce liver GCR expression, as it has been previously demonstrated in clinical and preclinical models of sepsis.^28^ *Gcr* knockdown in mice has also been shown to further exacerbate sepsis-induced liver injury, inflammation, and compromised liver function.^28^ GCR function and expression can also be regulated by heat shock proteins by facilitating ligand binding, protein stability, and preventing aggregation.^29^ Importantly, our study identified a reduction in the HSF1 pathway in AH-NR but not in AH-R, suggesting this may be a contributing factor to reduced GCR function and expression in AH-NR. Further, we found that AH-NR had higher levels of DHI1, the enzyme involved in the reversible metabolism of prednisolone to the less active prednisone.^30^ Since all nonresponders were treated with prednisolone and had reduced GCR expression, it is plausible that higher DHI1 would result in further compromised GCR signaling due to lower prednisolone levels. Recently, AZD4017 (a DHI1 inhibitor) was found to maintain prednisolone effectiveness without adverse effects in a safety and tolerability study in humans.^31^

It is well documented that AH-NR has a higher mortality rate relative to AH-R,^8^ for which the underlying mechanisms are currently being investigated. One study found that circulating hepatocyte-derived microvesicles were elevated in AH-NR versus AH-R and associated with enhanced mortality.^32^ These microvesicles from patients with AH stimulated immune cell TNFα production, possibly contributing to disease severity.^32^ Another study identified gene signatures of AH-NR versus AH-R through RNASeq analysis of PBMCs, including pathways involved in activation/proliferation and differentiation of T, B cells, and Natural killer cells and the mitochondrial electron transport chain.^33,34^ In our study, we aimed to identify changes in hepatic proteins associated with increased mortality in AH-NR. Decreased baseline APPs such as alpha-1-antichymotrypsin (anti-inflammatory),^35^ alpha-1-acid glycoprotein 2 (steroid carrier),^36,37^ and alpha-1-microglobulin (antioxidant)^38,39^ were found in patients who did not respond to therapy and did not survive to 24 weeks (AH-NR-NS) as compared to survivors (AH-NR-S). Lower levels of APPs at baseline in AH-NR-NS may contribute to the loss of anti-inflammatory and antioxidant functions of these APPs, increasing the likelihood of mortality. Another important observation was reduced levels of the coagulation factors coagulation factor X, kininogen-1, and plasminogen in AH-NR-NS relative to AH-NR-S. Coagulopathy is a hallmark of AH,^4^ and corticosteroids have been demonstrated to improve blood coagulation in patients with severe AH.^40^ Since AH-NR-NS had lower baseline expression of coagulation factors, this could contribute to gastrointestinal bleeding often seen in patients with AH^5^ and is likely a contributor to the increased mortality in these patients. We also found elevated markers of hepatic mast cells (chymase, tryptase alpha/beta 1, and carboxypeptidase A) in patients with AH-NR-NS . Mast cells have recently been demonstrated to exacerbate liver fibrogenesis^27,41^ through the release of pro-fibrotic factors.^42^ Of note, there are several Food and Drug Administration-approved mast cell stabilizers, such as cromolyn, that prevent degranulation and release of intracellular contents and have shown therapeutic efficacy in preclinical animal models of liver fibrosis.^43^ In addition, mast cells are involved in coagulopathy^44,45^ as they produce and release heparin,^44^ which may further compromise blood coagulation in patients with AH-NR and exacerbate gastrointestinal bleeding.

While this study is unique, several limitations need to be considered while interpreting the findings. First, the AH-R cohort consisted of patients that are younger than the AH-NR and the non-ALD controls. While AH-R are often younger than AH-NR,^8^ the AH-R versus control comparison is limited due to the significant age difference between the groups. Other confounding factors also exist, such as diet and genetics, but due to the limited available information, we were unable to control for these variables. Additional limitations of the study included the small sample size, and that the patient cohort consisted primarily of men. Since, the sample size in AH-NR-S and AH-NR-NS groups was limited to n = 4 and n = 3, respectively, observations from our study need to be validated in a larger independent cohort. Next, in our proteomic analysis, we were unable to distinguish between GCR-α and GCR-β due to the lack of exclusive peptide sequence in the regions that distinguish these 2 isoforms. This is an important consideration that we intend to investigate in future studies since GCR-β has been shown to inhibit GCR activity.^46^ Finally, we acknowledge that the findings from this study, although important, are exploratory and require further investigation and validation.

In conclusion, this study identified potential mechanisms/pathways contributing to a lack of response to corticosteroid treatment in patients with AH-NR, as well as changes in the liver protein landscape that may influence AH-NR mortality. These findings pave the way for future studies that may help to improve the standard treatment and care for patients with AH.

## Supplementary Material
