# Supplementary material for: Steroid responsiveness in alcohol-associated hepatitis is linked to glucocorticoid metabolism, mitochondrial repair, and heat shock proteins
Source: Hepatol Commun. 2024 Mar 4;8(3):e0393. doi: 10.1097/HC9.0000000000000393 (PMC10914234; doi:10.1097/HC9.0000000000000393)
Supplement: Supplementary file 1 [file hc9-8-e0393-s001.pdf]

Supporting Information

Hepatic protein differences between patients with alcohol-associated hepatitis who responded to corticosteroids vs. those who did not

Short title: Proteomic analysis of AH responders

Authors: Josiah Hardesty[1,2], Meghan Hawthorne[1], Le Day[3], Jeffrey Warner[1,2], Dennis Warner[1], Marina Gritsenko[3], Aliya Asghar[4], Andrew Stolz[5], Timothy Morgan[4], Craig McClain[1,2,6,7,8], Jon Jacobs[3], Irina Kirpich [2,7,8,9*]

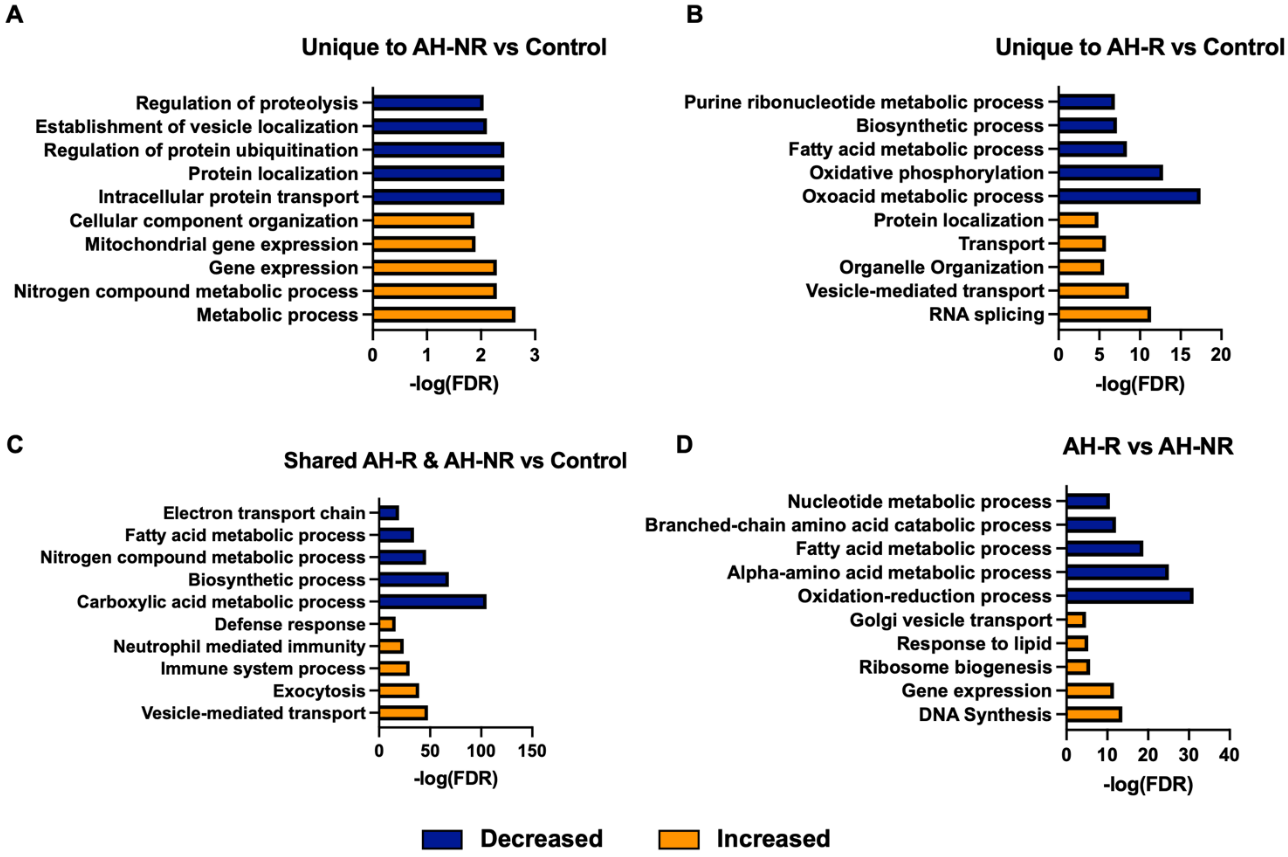

Figure S1: Hepatic processes altered in AH-R and AH-NR versus non-ALD controls. A. Gene ontology biological processes (GOBP) unique to AH-NR vs controls that are increased (gold) and decreased (blue). **B.** GOBPs unique to AH-R vs controls that are increased (gold) and decreased (blue). **C.** GOBPs shared between AH-NR and AH-R relative to controls that are increased (gold) and decreased (blue). **D.** GOBPs increased (gold) and decreased (blue) in AH-NR vs AH-R.

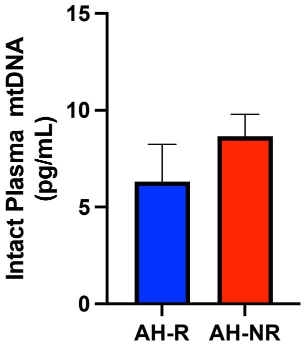

Figure S2: Intact mtDNA in AH-R and AH-NR. No differences were found in matched plasma intact mtDNA between AH-R (n=8) and AH-NR (n=4). Data are presented as mean □ SEM.
